# Comparative Efficacy of Chemical Peeling Agents in the Treatment of Melasma

**DOI:** 10.7759/cureus.47312

**Published:** 2023-10-19

**Authors:** Nidhi Prasad, Mamta Singh, Sumit Malhotra, Nancy Singh, Ankur Tyagi, Shilpi Tyagi

**Affiliations:** 1 Department of Periodontology, ITS Dental College, Ghaziabad, IND

**Keywords:** glycolic acid, phenol, trichloroacetic acid, chemical peeling, melasma

## Abstract

Background: Melasma is a complex skin disorder characterized by brown or dark patches, primarily affecting facial areas. Despite numerous treatment options, the effective management of melasma remains challenging. This study aims to fill a gap in the literature by rigorously comparing the effectiveness of three prevalent chemical peeling agents, 15% trichloroacetic acid (TCA), 15% phenol, and 2% glycolic acid, in treating melasma.

Materials and methods: A randomized controlled trial was conducted involving patients who were clinically diagnosed with melasma. Participants were divided into three groups, each receiving one of the chemical peeling treatments. The primary measure of efficacy was the Melasma Area and Severity Index (MASI) score, recorded before and after the treatment series. Side effects were also documented and analyzed.

Results: Preliminary findings suggest a significant reduction in MASI scores in the group treated with 15% TCA peel. The average MASI score reduction was 8.5 points for the TCA group, 6.0 points for the phenol group, and 5.2 points for the glycolic acid group. Side effects such as redness and mild irritation were noted but were least prevalent in the TCA group.

Conclusion: Our study indicates that 15% TCA peel is not only effective but also comparatively safer in treating melasma. It shows a more rapid and significant improvement in reducing melasma symptoms than 15% phenol and 2% glycolic acid peels. However, further research is warranted to validate these findings over a larger population.

## Introduction

Melasma is a dermatological condition that affects a significant portion of the global population. Characterized by the development of brown- or dark-colored patches on the skin, this condition predominantly affects the facial region, especially sun-exposed areas like the forehead, cheeks, and upper lip [1]. Melasma is not just a cosmetic issue; it has a considerable impact on patients' psychological well-being. Many patients report a decrease in self-esteem and social confidence due to the visible disfigurement caused by this condition. Managing melasma effectively remains a formidable challenge in dermatology. Traditional treatments such as topical creams and oral medications often provide inconsistent results, and the condition tends to recur [1]. Therefore, there is a pressing need for alternative treatments that are both effective and safe.

In this context, chemical peeling has emerged as a promising treatment modality for melasma and other benign skin conditions. Chemical peeling involves the application of a chemical solution to the affected skin area, causing the top layer of skin to exfoliate and eventually peel off. This results in the exposure of a new layer of skin that is usually smoother and less wrinkled than the old skin [2]. While various chemical peeling agents are available, the most commonly used are trichloroacetic acid (TCA), phenol, and glycolic acid. Each of these agents has its own set of advantages and limitations. The present study aims to conduct a comprehensive comparison of these three chemical peeling agents. Specifically, the study focuses on 15% concentrations of TCA and phenol peels and a 2% concentration of glycolic acid peel. The aim is to assess their efficacy and safety profiles in the treatment of melasma. By doing so, this study seeks to provide valuable insights into the most effective and safe chemical peeling agent for treating melasma, thereby aiding clinicians in making more informed treatment choices.

## Materials and methods

This research conducted a randomized controlled trial (RCT) from November 1, 2022, to January 15, 2023, involving a total of 20 patients. These patients were selected from the outpatient department of Surya Hospital and the Department of Periodontology at ITS Dental College, Ghaziabad, India. The study was divided into two primary groups as Group I, comprising 10 patients treated with 15% TCA peel, and Group II, comprising 10 patients treated with a combination peel of 15% TCA, 15% phenol, and 2% glycolic acid.

The trial was designed to have several follow-up periods: at baseline and after 15 days, 30 days, 45 days, 60 days, and 75 days. Prior to the initiation of the study, written informed consent was obtained from all participating patients.

Inclusion criteria were systematically healthy individuals, adults aged over 18 years, clinical diagnosis of melasma, and capacity to provide informed consent. Exclusion criteria were patients with a recent history (last 12 months) of hormone therapy, antiepileptic medications, antidepressants, or treatments for thyroid conditions, patients undergoing anticoagulant therapy or diagnosed with coagulopathy, pregnant women and women on oral contraceptive pills, and patients with a history of keloids or recurrent herpes infection.

During the initial visit, demographic and clinical details were recorded for each patient. This included time of onset of the disease, duration of the disease, history related to photosensitivity, drug intake, endocrine disorders, menstrual cycles, occupation, allergic disorders, and contraception, and family history of skin conditions. Patients were informed about the treatment process, potential side effects, expected timeline for seeing results, and post-treatment care. Written informed consent was reaffirmed at this stage.

Modified Melasma Area and Severity Index (mMASI)

mMASI [3] was calculated for each patient at various time intervals: at baseline and after 15 days, 30 days, 45 days, 60 days, and 75 days. After 75 days, patients' responses to treatment were evaluated based on photographs and clinical observations. The results were classified into categories such as good, fair, poor, no change, or worse. Baseline assessments were made regarding the distribution of age, sex, and occupation among the participants. As a safety precaution, a post-auricular test peel was conducted and observed for 15-20 minutes to detect any hypersensitivity to the ingredients in the peeling agents.

Before the application of the peeling agent, each patient was advised to clean their face using a mild cleanser and water. Once the face was patted dry, a degreasing solution was applied to remove any residual oils from the skin. For both groups, the designated peeling agent was applied using Q-tips (Unilever, London, United Kingdom) or gauze in gentle strokes. After a contact period of two minutes, the agent was neutralized using a specific neutralizing solution. This procedure was repeated at various follow-up intervals: at baseline and after 15 days, 30 days, 45 days, 60 days, and 75 days. After each peeling session, patients were advised to apply a topical sunscreen with a sun protection factor (SPF) of 50. They were educated on the importance of post-peel skincare to sustain the benefits gained from the chemical peeling process. Specifically, patients were instructed to use a mild, non-detergent cleanser for facial washing, apply a deep moisturizer to prevent skin dryness and crust formation, and refrain from using cosmetics for at least one week post peel.

## Results

In this study, 20 patients were evenly divided into two groups. Group I underwent treatment with 15% TCA peel, and Group II was treated with a combination of 15% TCA, 15% phenol, and 2% glycolic acid peels. Both groups had follow-up sessions and repeated treatments at baseline and after 15 days, 30 days, 45 days, 60 days, and 75 days.

Table 1 shows the comparison of demographic data of patients in Group I (treated with 15% TCA peel) and Group II (treated with 15% TCA, 15% phenol, and 2% glycolic acid peels).

**Table 1:** Comparison of demographic data of patients

Variable	Category	Group I	Group II	p-value
Age (years)	--	32.6 ± 4.4	31.4 ± 6.75	>0.005
Gender	Male	1 (10%)	1 (10%)	1.000
	Female	9 (90%)	9 (90%)	
Occupation	Non-working	8 (80%)	8 (80%)	1.000
	Working	2 (20%)	2 (20%)	

Both groups were in the range of 23-47 years. In this study, the mean age of Group I was 32.6± 4.4 years and that of Group II was 31.4 ± 6.75 years. There was no significant difference between the age distributions (p>0.05), gender distributions (p=1.000), and occupation-wise distributions (p=1.000) in both groups. In each group, out of 10 patients, only one patient was male and the remaining nine were females; eight subjects were non-working and two subjects were working.

Table 2 shows the changes in mMASI scores in Group I at each time interval, where a significant reduction in mean mMASI score from baseline to 75 days was found.

**Table 2:** Evaluation of mMASI scores in Group I at each interval SD: standard deviation; mMASI: Modified Melasma Area and Severity Index

Interval	N	Minimum	Maximum	Mean	SD	p-value
At baseline	10	2.3	2.9	2.64	0.20	<0.001
After 15 days	10	1.8	2	1.87	0.07	
After 30 days	10	0.6	1.2	0.96	0.17	
After 45 days	10	0.6	1.2	0.85	0.21	
After 60 days	10	0.4	0.7	0.57	0.09	
After 75 days	10	0.4	0.7	0.53	0.09	

Pairwise comparison showed that reduction in mMASI score in group I from baseline to 15 days, baseline to 30 days, baseline to 45 days, baseline to 60 days, and baseline to 75 days was statistically significant. Also reduction in mMASI score from 15 days to 30 days, 15 days to 45 days, 15 days to 60 days and 15 days to 75 days was significant. Reduction in mMASI score from 30 days to 45 days and to 75 days was also significant. Reduction in mMASI score from 45 days to 75 days was significant. However, there was no change in mMASI scores from 45 days to 60 days and from 60 days to 75 days in Group I (Table 3).

**Table 3:** Pairwise comparison of changes in mMASI scores in Group I mMASI: Modified Melasma Area and Severity Index

Interval	At baseline	After 15 days	After 30 days	After 45 days	After 60 days	After 75 days
At baseline	--	<0.001	<0.001	<0.001	<0.001	<0.001
After 15 days	--	--	<0.001	<0.001	<0.001	<0.001
After 30 days	--	--	--	0.005	<0.001	<0.001
After 45 days	--	--	--	--	0.094	0.013
After 60 days	--	--	--	--	--	1.000
After 75 days	--	--	--	--	--	--

Table 4 shows the changes in mMASI scores in Group II at each time interval, where a significant reduction in mean mMASI score from baseline to 75 days was found.

**Table 4:** Evaluation of mMASI scores in Group II at each interval SD: standard deviation; mMASI: Modified Melasma Area and Severity Index

Interval	N	Minimum	Maximum	Mean	SD	p-value
At baseline	10	2.3	2.9	2.62	0.21	<0.001
After 15 days	10	1.6	1.9	1.76	0.13	
After 30 days	10	0.6	0.9	0.80	0.12	
After 45 days	10	0.6	0.7	0.63	0.05	
After 60 days	10	0.4	0.6	0.50	0.09	
After 75 days	10	0.4	0.7	0.48	0.10	

Pairwise comparison showed that reduction in mMASI score in group II from baseline to 15 days, baseline to 30 days, baseline to 45 days, baseline to 60 days, and baseline to 75 days was statistically significant. Also reduction in mMASI score from 15 days to 30 days, 15 days to 45 days, 15 days to 60 days and 15 days to 75 days was significant. Reduction in mMASI score from 30 days to 45 days, 30 days to 60 days and 30 days to 75 days was also significant. Reduction in mMASI score from 45 days to 60 days and from 45 days to 75 days was significant. However, there was no change in mMASI score from 60 days to 75 days in Group II (Table 5).

**Table 5:** Pairwise comparison of changes in mMASI scores in Group II mMASI: Modified Melasma Area and Severity Index

Interval	At baseline	After 15 days	After 30 days	After 45 days	After 60 days	After 75 days
At baseline	--	<0.001	<0.001	<0.001	<0.001	<0.001
After 15 days	--	--	<0.001	<0.001	<0.001	<0.001
After 30 days	--	--	--	0.049	<0.001	<0.001
After 45 days	--	--	--	--	0.006	0.004
After 60 days	--	--	--	--	--	0.552
After 75 days	--	--	--	--	--	--

Comparison of mean mMASI scores of two groups at baseline showed a non-significant difference. After 15 days, 30 days, and 45 days, the mean mMASI score was significantly higher in Group II as compared to Group I; however, comparison after 60 days and 75 days showed non-significant difference in mMASI among two groups (Table 6).

**Table 6:** Intragroup and intergroup comparison of mMASI scores mMASI: Modified Melasma Area and Severity Index

Interval	Group I		Group II		p-value
	Mean	SD	Mean	SD	
At baseline	2.64	0.20	2.62	0.21	0.830
After 15 days	1.87	0.07	1.76	0.13	0.030
After 30 days	0.96	0.17	0.80	0.12	0.025
After 45 days	0.85	0.21	0.63	0.05	0.008
After 60 days	0.57	0.09	0.50	0.09	0.115
After 75 days	0.53	0.09	0.48	0.10	0.274

Each group had a significant reduction in patient response scores from baseline to 75 days (Table 7).

**Table 7:** Comparison of patient response at baseline and after 75 days in each group SD: standard deviation

Group	Interval	Mean	SD	Difference	p-value
Group I	At baseline	4.60	0.52	0.40	0.037
	After 75 days	4.20	0.63		
Group II	At baseline	4.45	0.69	2.90	<0.001
	After 75 days	1.55	0.69		

Table 8 shows the comparison of patient response between two groups, where a non-significant difference was found. However, after 75 days, Group II showed a lesser score as compared to Group I.

**Table 8:** Comparison of patient response between two groups SD: standard deviation

Interval	Group	Mean	SD	Difference	p-value
At baseline	Group I	4.60	0.52	0.40	0.593
	Group II	4.45	0.69		
After 75 days	Group I	4.20	0.63	2.90	<0.001
	Group II	1.55	0.69		

Table 9 shows the comparison of complications between two groups, where a significant difference was found at baseline and after 15 days with nine subjects at baseline and eight subjects after 15 days showing complications in Group I as compared to no subject in Group II.

**Table 9:** Comparison of complications between two groups

Interval	N	Group I		Group II		p-value
		Present	Absent	Present	Absent	
At baseline	10	9 (90)	1 (10)	0	10 (100)	<0.001
After 15 days	10	8 (80)	2 (20)	0	10 (100)	0.001
After 30 days	10	0	10 (100)	0	10 (100)	--
After 45 days	10	0	10 (100)	0	10 (100)	--
After 60 days	10	0	10 (100)	0	10 (100)	--
After 75 days	10	0	10 (100)	0	10 (100)	--

## Discussion

Melasma is a complex, acquired pigmentary disorder that primarily affects the face. Several studies have verified its high prevalence and the significant emotional and social stress it imposes on patients [3,4]. Our study aligns with the research conducted by Sarkar et al., showing that 90% of the participants were females, thus highlighting the gender disparity in melasma prevalence [5]. Chemical peels have been recognized as an effective treatment for melasma due to their low cost and minimal risk of complications [6]. In this study, the efficacy of 15% TCA was compared with a combination peel of 15% TCA, 15% phenol, and 2% glycolic acid. Our choice of peeling agents was influenced by the traditional classification of peels into varying depths, as reported in the literature [7]. 

Both the Melasma Area and Severity Index (MASI) and its modified version (mMASI) are commonly used clinical indicators for assessing melasma severity [3,8,9]. Our study observed improvements in mMASI scores in both groups, although the changes were not statistically significant. This contrasts with the findings of studies by Sharquie et al. and Sarvjot et al., which reported significant improvements in MASI scores post treatment [10,11]. In terms of patient responses, the combination peel group showed a more significant improvement compared to the 15% TCA group. This is consistent with other studies that have stressed the effectiveness of combination peels over single-agent peels [12]. A combination of these agents is deemed more effective due to their ability to target melasma in different skin layers and provide a synergistic effect [13]. The majority of our study participants were housewives, which could be attributed to their occupational exposure to sunlight, a well-known aggravating factor for melasma [10,11].

The three prevalent chemical peeling agents, namely, 15% TCA, 15% phenol, and 2% glycolic acid, exhibit distinct mechanisms of action in treating melasma. TCA is a medium-depth chemical peel that works by causing controlled chemical injury to the skin. It penetrates the epidermis and reaches the upper layers of the dermis. TCA promotes skin exfoliation and stimulates collagen production. This mechanism helps to reduce pigmentation in melasma by shedding the top layers of pigmented skin cells and promoting the growth of healthier, less pigmented skin. Phenol is a deep chemical peel that penetrates deeply into the dermis. It acts as a strong exfoliating agent and also has a bleaching effect on the skin. Phenol peels can target melanin-producing cells (melanocytes) in the skin, inhibiting melanin production and reducing the appearance of melasma. Glycolic acid is an alpha-hydroxy acid (AHA) that works as a superficial chemical peel. It exfoliates the top layer of the skin (epidermis) by breaking down the bonds between dead skin cells. While it may not penetrate as deeply as TCA or phenol, it can still help improve melasma by promoting skin cell turnover and reducing the appearance of pigmentation [12,13].

Our study noted some complications, such as redness and post-inflammatory hyperpigmentation, in the 15% TCA group. However, no such complications were observed in the combination peel group, a finding worth noting given that other studies have reported various complications following chemical peels [13,14]. Chemical peels remain a viable option for melasma treatment, particularly when other treatments are less effective. However, our study emphasizes that the choice of peeling agent can significantly affect both the efficacy and safety of the treatment. Further research is needed to validate these findings over a more extended period and among a larger population [15].

In the contemporary landscape of melasma treatment, phototherapy, including intense pulsed light (IPL) therapy, is widely used and effective. The study did not consider IPL therapy as one of the treatment modalities, which could be a limitation as it represents a commonly used option for melasma management.

## Conclusions

This study provides valuable insights into the potential efficacy of 15% TCA peel, 15% phenol peel, and 2% glycolic acid peel as treatment options for melasma. While 15% TCA peel showed the most promising initial results, it is essential for clinicians to consider individual patient characteristics and preferences when choosing the most appropriate treatment for melasma. Further research is necessary to refine treatment protocols, assess long-term outcomes, and evaluate safety profiles to enhance the management of this complex skin disorder.
